# Non-selective Day-1 Haemoglobin Monitoring Following Elective Primary Knee Arthroplasty: Clinical Value or Outdated Practice?

**DOI:** 10.7759/cureus.93588

**Published:** 2025-09-30

**Authors:** Abdulrahman Kashkosh, Bawan Hama, Abhishek Vashista, Syed Y Ahmad, Ahsan M Sani, Ganapathy R Perianayagam, Girish Vashista

**Affiliations:** 1 Trauma and Orthopaedics, Doncaster and Bassetlaw Teaching Hospitals NHS Foundation Trust, Doncaster, GBR; 2 Trauma and Orthopaedics, Bradford Teaching Hospitals NHS Foundation Trust, Bradford, GBR; 3 School of Medicine and Population Health, University of Sheffield, Sheffield, GBR; 4 Intensive Care Unit, St George's University Hospitals NHS Foundation Trust, London, GBR; 5 Geriatric General Medicine, Manchester University NHS Foundation Trust, Manchester, GBR

**Keywords:** blood conservation, knee arthroplasty, postoperative haemoglobin monitoring, selective testing, tranexamic acid

## Abstract

Background

Knee arthroplasty is one of the most commonly performed elective surgeries in the UK. Routine Day-1 postoperative haemoglobin (Hb) monitoring is standard practice, but its value is increasingly questioned. Improvements in perioperative care, surgical technique, and the use of adjuncts have significantly reduced blood loss and transfusion rates.

Objectives

The main objective of this study is to evaluate the need for universal, routine Day-1 Hb monitoring following elective primary knee arthroplasty by assessing the mean Hb drop, the rate of severe anaemia, and the rate of red blood cell (RBC) transfusions.

Methods

We conducted a retrospective audit of 452 consecutive total and unicondylar primary knee arthroplasties performed by a single surgeon between March 2013 and February 2020. Standardised surgical techniques were employed, including an anteromedial approach, universal tranexamic acid (TXA) and adrenaline administration, avoidance of surgical drains, controlled blood pressure management, and electrocautery haemostasis. A restrictive transfusion protocol was followed (transfusion for symptomatic anaemia or Hb <70 g/L). The primary outcomes were the mean Hb drop, the incidence of severe anaemia, and RBC transfusion rates.

Results

Mean preoperative Hb was 137.2 ± 14.2 g/L, decreasing to 113.8 ± 14.1 g/L on Day 1 (mean drop: 23.3 ± 9.0 g/L). Only six patients (1.3%) had Day-1 Hb levels below 80 g/L, and 12 patients (2.4%) required postoperative transfusion. No patients were required to return to theatre for haemorrhage control, and no transfusion-related complications occurred.

Conclusions

With modern blood conservation techniques, universal Day-1 Hb monitoring offers minimal clinical value in elective primary knee arthroplasty. We recommend selective Hb testing for patients with low preoperative Hb levels, relevant comorbidities, or clinical symptoms of anaemia, rather than routine testing for all patients. This approach maintains patient safety while reducing unnecessary interventions, healthcare costs, and environmental impact.

## Introduction

Primary knee arthroplasty, both total and unicondylar, represents one of the most commonly and successfully performed orthopaedic surgeries in the UK and worldwide [1]. For patients with advanced osteoarthritis, this operation significantly improves their quality of life by relieving pain and improving knee joint function. Moreover, the demand for knee arthroplasty has grown rapidly, driven primarily by an ageing population. In 2022, England and Wales had an estimated 103,000 primary knee replacements performed, with numbers continuing to rise and expected to exceed the pre-pandemic peak of approximately 110,000 per year, particularly in light of government plans for surgical hubs to address the long waiting lists [2,3]. Similarly, in the US, Medicare data projects that by 2040, 1.2 million knee replacements per year will be expected [4]. Given this substantial increase in surgical volumes, it is therefore essential that robust techniques and protocols are available, regularly audited, and improved. In response to these demands, modern knee arthroplasties have undergone substantial advancements in their designs, surgical technique, adjunct use, and perioperative care [5].

Perioperative haemoglobin (Hb) optimisation is fundamental in surgical practice. In arthroplasty, this has improved significantly, owing to evidence-based blood conservation protocols. These protocols include the use of tranexamic acid (TXA), local infiltration of adrenaline, electrocautery, and careful intraoperative blood pressure management, which means that, although knee replacements remain major surgery, significant haemorrhage and red blood cell (RBC) transfusions are now rare [6].

Nevertheless, most UK units continue to perform routine Hb testing on postoperative Day 1 after elective knee arthroplasty. This practice is based on the rationale of screening for occult bleeding or anaemia. The National Institute for Health and Care Excellence (NICE) guidelines recommend transfusions for symptomatic anaemia or when Hb levels fall below 70 g/L in stable patients [7].

However, recent studies suggest that indiscriminate Day-1 Hb checks rarely change the management of this patient population. Meanwhile, unnecessary blood testing has several downsides, including extended hospital stays and significant financial and environmental costs [8].

Therefore, this audit was designed to evaluate the clinical value of routine Day-1 Hb testing at our centre. Our review analysed seven years of elective knee arthroplasty data from a high-volume surgeon, focusing specifically on Hb drop, severe anaemia, and transfusion rates. We interpreted our findings in light of recent literature on postoperative Hb monitoring, discussing the advantages and disadvantages of universal Hb monitoring, current blood loss reduction strategies, and the potential of a selective approach to Hb monitoring. 

## Materials and methods

We conducted a retrospective audit of consecutive elective primary knee arthroplasties performed by a single consultant surgeon at Doncaster and Bassetlaw Teaching Hospitals NHS Foundation Trust, Doncaster, England, between March 2013 and February 2020. Our inclusion criteria encompassed adult patients undergoing primary total knee arthroplasty (TKA) and unicondylar knee arthroplasty (UKA), including “complex” primary cases. Conversely, the exclusion criteria included bilateral operations, revision surgeries - such as unicondylar to total knee surgery - and cases with missing data on pre- or postoperative Hb levels. A total of 452 cases met the inclusion and exclusion criteria and were included in the final analysis. 

Standard operative practices were followed for all procedures. General or spinal anaesthesia was administered according to the standing practice protocols, whilst systolic blood pressure was maintained between 90 and 100 mmHg intraoperatively. Moreover, standardised blood conservation measures were consistently implemented across all patients. Specifically, all patients received 1 g of intravenous TXA at induction, followed by another 1 g eight hours postoperatively. Tourniquets were applied universally. An anteromedial parapatellar approach was universally employed, and, subsequently, after making the bone cuts, bupivacaine with adrenaline was infiltrated for local haemostasis. Intraoperatively, haemostasis was achieved with electrocautery throughout the procedure. Low molecular weight heparin was given six hours postoperatively, and, importantly, no surgical drains were placed in any case. 

Hb monitoring was conducted systematically, with all cases having preoperative Hb levels measured on admission and subsequent monitoring on the morning of postoperative Day 1, approximately 18 to 24 hours post-recovery. Furthermore, the indications for RBC transfusion were standardised according to NICE guidelines, whereby transfusion was only indicated if Hb levels fell below 70 g/L, or in cases of symptomatic anaemia presenting with symptoms such as tachycardia, hypotension, or other clinical signs of anaemia confirmed by a low Hb count. When transfusion was required, patients underwent post-transfusion Hb monitoring to confirm treatment effectiveness. 

Patient data were recorded electronically using the Integrated Clinical Environment (ICE) system, capturing preoperative and Day-1 Hb levels, and any perioperative transfusions administered either intraoperatively or postoperatively. The primary outcomes assessed were the mean drop in Hb from preoperative levels to Day 1, the incidence of severe postoperative anaemia, defined as Hb < 80 g/L, and the overall rate of RBC transfusion. This audit was presented at the Clinical Governance Department at Doncaster and Bassetlaw Teaching Hospitals NHS Foundation Trust, and formal ethical approval was not required, as this constituted a retrospective audit of routine clinical practice. 

## Results

We investigated 452 elective primary knee arthroplasties, comprising 432 TKAs and 20 UKAs.

Preoperatively, the mean Hb level was 137.2 g/L (±14.2). On postoperative Day 1, the mean Hb decreased to 113.8 g/L (±14.1), representing a mean drop of 23.3 g/L (±9.0) from baseline. Importantly, only six patients (1.3%) had a Day-1 Hb below 80 g/L, which represents our severe anaemia threshold. In total, 12 patients (2.4%) received RBC transfusions during their hospitalisation. Notably, no patient required a return to the operating theatre for haemorrhage control, and no transfusion reactions or infections from transfused blood were observed throughout the audit period (Table 1).

**Table 1:** Haemoglobin Levels and Transfusion Outcomes in 452 Primary Knee Arthroplasty Cases

Outcome	Result
Preoperative haemoglobin, mean ± SD (g/L)	137.2 ± 14.2
Postoperative Day-1 haemoglobin, mean ± SD (g/L)	113.8 ± 14.1
Mean haemoglobin drop ± SD (g/L)	23.3 ± 9.0
Patients with postoperative Hb < 80 g/L, n (%)	6 patients (1.3%)
Patients requiring transfusion, n (%)	12 patients (2.4%)

## Discussion

Our audit confirms that routine Hb monitoring on postoperative Day 1 yields little actionable information for nearly all primary knee arthroplasty patients in the modern surgical setting. As shown in Table 1, severe postoperative anaemia was uncommon, occurring in only 1.3% of cases.

Our audit findings align closely with those of the most recent studies in this field. Hennessy and McSorley (2020) found that only five patients (3.7%) out of 136 post-arthroplasty cases had a Day-1 Hb low enough to warrant intervention [9]. Similarly, Howgate et al. (2024) reported only a 0.5% transfusion rate in 200 TKA patients [10]. Both studies noted that Day 1 postoperative Hb tests did not influence clinical management decisions. However, some modern studies have reported higher intervention rates. Nuñez et al. (2022) found transfusion rates of 9.1% for TKA and 2% for UKA, though these differences may reflect variations in patient populations, blood conservation, and transfusion protocols [11]. 

Vestermark et al., in a 2022 study of 682 TKA cases, found that only 1.1% required blood transfusion overall and attributed this low rate to the routine use of TXA [12]. Gilde et al. (2021) further supported this, adding that when TXA is used and preoperative Hb is greater than 12 g/dL, routine Hb testing becomes obsolete [13]. 

The impact of modern blood conservation techniques is particularly evident when historical data are compared. Studies conducted before the routine use of TXA often reported transfusion rates exceeding 10%. Bedard et al. (2017), in a large multicentre study examining nearly 140,000 total knee replacements, demonstrated that the need for blood transfusion decreased from 17.3% to 4.4% over the years 2007-2015 [14]. 

Several measures we used are known to reduce blood loss and can be attributed to our low severe anaemia and transfusion rates through the reduction of both occult and visible bleeding. These advances in blood-sparing techniques include electrocautery haemostasis, avoidance of drains to prevent rebound bleeding, controlled hypotensive anaesthesia, and the universal use of TXA intraoperatively and eight hours postoperatively [15]. 

Emphasising proven blood loss conservation techniques is paramount. TXA is now considered standard practice in most knee arthroplasty units. Additionally, the use of TXA is in accordance with NICE guidelines [7]. Robust data from a 2021 meta-analysis demonstrate that TXA dramatically reduces perioperative haemorrhage and transfusion requirements without raising the risks of venous thromboembolism (VTE) or myocardial infarction [16]. In our series, we used TXA in every patient and observed no thrombotic complications. 

Another important measure is to avoid the use of surgical drains. Studies have indicated that the use of drains does not reduce blood loss and may aggravate postoperative bleeding [17]. We believe that our low RBC transfusion rate partly reflects the routine implementation of evidence-based protocols.

Disadvantages of universal day-1 Hb checks 

Reducing "fishing expeditions" or over-investigation is an important principle in modern medicine. Each venepuncture can cause patient discomfort, anxiety, bruising, and, rarely, iatrogenic complications. Whilst the blood volume lost during a single venepuncture is minimal - typically 10 mL - and the risk of worsening anaemia through repeated testing is small, the key concern is that these results may bias transfusion decisions. It has been observed in other specialties that routine Hb monitoring can paradoxically lead to higher transfusion rates without improving patient outcomes [18,19]. 

Therefore, avoiding routine testing of postoperative Hb may reduce the likelihood of unnecessary transfusions. This is notably significant, as RBC transfusion carries well-known risks, including immune reactions such as haemolytic and non-haemolytic transfusion reactions, and transmission of blood-borne viruses, including HIV and hepatitis B and C. Additional complications include transfusion-associated circulatory overload (TACO) and transfusion-related acute lung injury (TRALI), with these risks being particularly elevated in older adults, who constitute the predominant knee arthroplasty patient demographic. Studies have also discussed the specific risks related to transfusion in arthroplasty, including higher infection rates and increased morbidity. Additionally, prolonged inpatient admissions increase patient exposure to hospital-acquired infections and delirium [20,21]. 

Whilst patient care remains the primary consideration, institutional factors warrant consideration. Routine Hb testing incurs costs and resources that must be evaluated. Routine venepuncture and laboratory analyses consume significant resources, including the time of phlebotomists, nursing teams, laboratory technicians, and associated processing costs. A recent systematic review estimated that eliminating universal blood testing post-arthroplasty could save thousands of pounds per centre annually [6]. 

Additionally, environmental considerations, particularly in the context of developing "green" orthopaedic theatres, have become increasingly relevant. Each omitted blood test contributes to reduced disposal of plastics and associated environmental impacts. For example, a basic full blood count (FBC) and urea and electrolytes (U&Es) test generate approximately 332 g CO₂ equivalent [22]. Consequently, routine Hb monitoring leads to avoidable greenhouse gas emissions. 

Notably, healthcare systems globally are burdened by financial and operational pressures. Eliminating unnecessary tests represents an important opportunity to reduce these pressures and optimize resource allocation. Importantly, in the current UK healthcare climate, with pressure on hospital bed availability, post-operative testing may delay discharge; this is also a significant consideration, as many units are pursuing day-case pathways [23]. 

Our audit, aligned with recent studies, found that 97.6% of patients never required RBC transfusion despite Hb monitoring, demonstrating that nearly all tests were wasteful. Thus, when clinical, financial, operational, and environmental factors are considered, the evidence strongly supports adopting selective testing in the post-elective arthroplasty context.

Case for routine testing 

Conversely, one can argue that, with a mean Hb drop of 23.3 g/L, routine Day-1 Hb monitoring can ensure early detection of any unexpected bleeding. Thus, once a fall in Hb beyond the expected parameters is observed, this could prompt active monitoring of patient symptoms and earlier intervention. 

Patients often experience postoperative fatigue and may develop light-headedness and postural hypotension. However, these symptoms can be easily dismissed as normal postsurgical sequelae, rather than being recognised as potential signs of significant anaemia. An objective Hb value could help eliminate this diagnostic uncertainty. Additionally, postoperative anaemia may be masked by the surgical stress response, making Hb monitoring essential for identifying true anaemia linked to adverse outcomes [24,25]. 

Additionally, it is common practice in the UK for post-knee arthroplasty patients to receive anticoagulation as VTE prophylaxis for 14 days. One could argue that having a postoperative Hb value ensures that patients are not starting anticoagulation with unrecognised anaemia, giving surgeons the confidence to prescribe and discharge patients on extended VTE prophylaxis. Furthermore, the patient cohort, predominantly elderly, may prefer to remain in hospital for observation after major surgery, and performing a blood test on Day 1 may be reassuring to both patient and surgeon [1,26]. 

Predicting patients who may need Hb monitoring

Ultimately, universal routine Day-1 postoperative Hb testing in elective knee arthroplasty seldom yields clinically actionable results, a limitation that is even more pronounced in low-risk patient groups. Gilde et al. (2021), Nuñez et al. (2022), and Angerame et al. (2021) all advocate for a more targeted testing approach, guided by baseline patient characteristics [11,13,27]. 

Angerame et al. (2021) concluded that routine pre- and postoperative laboratory tests for all patients were not necessary. They recommended focusing on patients with relevant comorbidities, particularly those with a history of hepatobiliary disease, arrhythmia, coronary artery disease, congestive heart failure, vascular or haematologic disease, chronic kidney disease, and insulin-dependent diabetes mellitus [27]. 

Nuñez et al. (2022) and Gilde et al. (2021) studies, which combine a cohort of around 2,000 patients, both conclude that pre-transfusion tests should only be performed for patients with preoperative Hb <12 g/dL, given that low preoperative Hb was the strongest predictor of requiring blood transfusions. Practically, this approach could translate into maintaining Day-1 Hb checks for patients who underwent surgery with low Hb levels, specific comorbidities, or in cases where intraoperative bleeding or surgical difficulties were encountered. For routine cases with normal preoperative Hb levels and uneventful surgery, clinical monitoring for symptoms or signs of anaemia would seem reasonable and sufficient. Nevertheless, it is important to consider that all three studies were retrospective and were limited by potential biases - especially recall bias and selection bias - making it harder to establish causality [11,13]. 

Finally, another important predictor is the American Society of Anesthesiologists (ASA) grade. A large retrospective study analysed 48,055 TKAs, finding that ASA Class 3-4 was an independent predictor of the need for blood transfusion. Thus, this physical status classification system can be a useful aid, providing a simple numerical score to identify patients who may need postoperative blood transfusion [28]. 

Limitations 

This audit had several important limitations that must be acknowledged. Most notably, it represents a single-surgeon, single-centre retrospective audit, which inherently limits the generalisability of our findings to other institutions and surgical practices. Our results reflect a centre with well-established blood conservation measures, and other units with different practice patterns or transfusion protocols might observe substantially different outcomes. Finally, as an observational audit, we cannot definitively prove that omitting Day-1 Hb monitoring would not have resulted in missed severe anaemia. However, the risk appears to be low under our current unit protocols.

## Conclusions

In this audit of elective primary knee arthroplasty, routine Day-1 Hb monitoring yielded minimal clinical benefit. Severe anaemia was very rare, occurring in only 1.3% of patients, and transfusions in only 2.4%. Given the progress in blood-conserving techniques, low transfusion rates, and the well-characterised disadvantages of overtesting, we conclude that universal routine Day-1 Hb monitoring can be safely omitted in arthroplasty units that have implemented comprehensive blood conservation measures. We propose that arthroplasty units should establish a selective approach that reserves Day-1 Hb checks for patients with low pre-operative Hb, specific risk factors, or those who develop symptomatic anaemia. This selective strategy would maintain patient safety whilst reducing unnecessary interventions, costs, and environmental impact.

In summary, non-selective Day-1 Hb testing after primary knee arthroplasty appears to be redundant in modern practice, and selective testing approaches are recommended to enhance clinical value. However, future research is needed to develop and validate accurate prediction tools that can reliably identify patients who may require RBC transfusion, and therefore warrant postoperative Hb monitoring. 
